# First person – Toru Miwa

**DOI:** 10.1242/bio.046821

**Published:** 2019-08-15

## Abstract

First Person is a series of interviews with the first authors of a selection of papers published in Biology Open, helping early-career researchers promote themselves alongside their papers. Toru Miwa is first author on ‘Role of Dach1 revealed using a novel inner ear-specific Dach1-knockdown mouse model’, published in BiO. Toru conducted the research described in this article while a post-doc fellow of ENT in University of Southern California, in Takahiro Ohyama's lab at the Keck School of Medicine, USA. He is now a head physician specializing in ENT at JCHO Kumamoto General Hospital, Japan, investigating the inner ear.


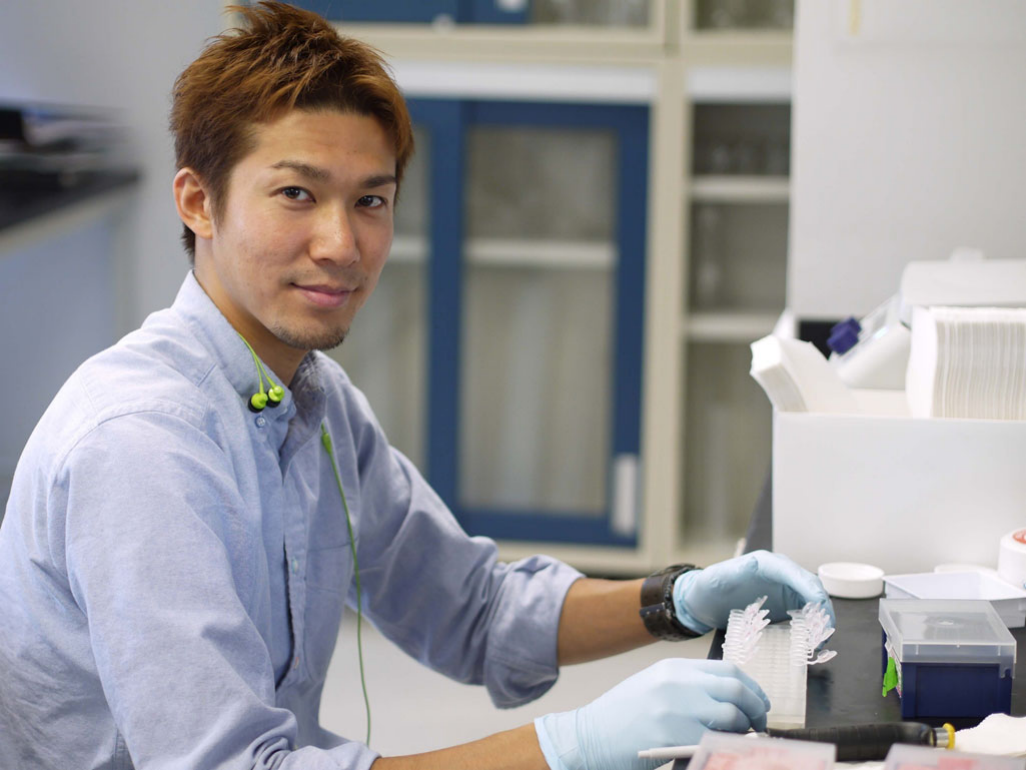


**Toru Miwa**

**What is your scientific background and the general focus of your lab?**

I am an otolaryngologist and researcher of inner ear biology. Seven years ago, I was finalizing my MD PhD thesis about embryonic gene therapy for congenital hearing loss utilizing electroporation-mediated transuterine gene transfer into otocysts (EUGO). The general focus of our lab is to investigate the molecular mechanisms involved in the development of the inner ear in order to find and characterize new aspects for developing the gene therapy for congenital hearing loss.

**How would you explain the main findings of your paper to non-scientific family and friends?**

I always answer this kind of question, ‘to develop a cure for hearing loss for patients all over the world’. Hearing loss is a common disease and many people struggle with it. However, the work I am doing in the lab, although at a very experimental level, aims to understand the mechanism of inner ear development and hopefully bring meaningful knowledge to the scientific community, especially when designing novel hearing therapies. The main finding of the current paper is that one molecule that we were looking at is involved in inner ear development and affects the surrounding gene(s), and in this way it may regulate the morphogenesis of the inner ear. This kind of basic research is fundamental for translating new discoveries into clinical use.

**What are the potential implications of these results for your field of research?**

I hope that these results bring more functional knowledge to the inner ear research field. The field itself is old and a number of great publications exist. However, I feel that more analysis of genes and proteins is necessary, especially about their cellular mechanisms. Our research will be useful in the future for diagnoses of congenital hearing loss. Moreover, they could be potential targets for novel therapy of hearing loss.

**What has surprised you the most while conducting your research?**

At start of my project, I recognized clearly how time consuming it is to do research. This manuscript is not just doing lab work and writing the results. It has been the most surprising to realize what it takes to be a researcher; a multitasking specialist in diverse fields. The longer I have been the researcher, the more confidence I have obtained.

“It has been the most surprising to realize what it takes to be a researcher; a multitasking specialist in diverse fields.”

**What, in your opinion, are some of the greatest achievements in your field and how has this influenced your research?**

I would say the paper in 2013, ‘Mouse otocyst transuterine gene transfer restores hearing in mice with connexin 30 deletion-associated hearing loss’, is the greatest achievement in my field and was our first published article about embryonic gene therapy based on our results. It is the first embryonic gene therapy for congenital hearing loss, thus it has definitely brought new insight about gene therapy. To me it has given me courage to continue with the research, as there are many more embryonic gene therapies to be discovered and investigated.

**What changes do you think could improve the professional lives of early-career scientists?**

I feel the decreasing amount of people who are performing scientific research in Japan, despite continuing high-quality research and making new discoveries all over the world. I consider financial support important for professional lives of early-career scientists. This is the issue that each scientist is struggling with, however, I hope for better access to early-career funding. Primarily, this is an issue to be solved at the governmental level.

**Figure:**
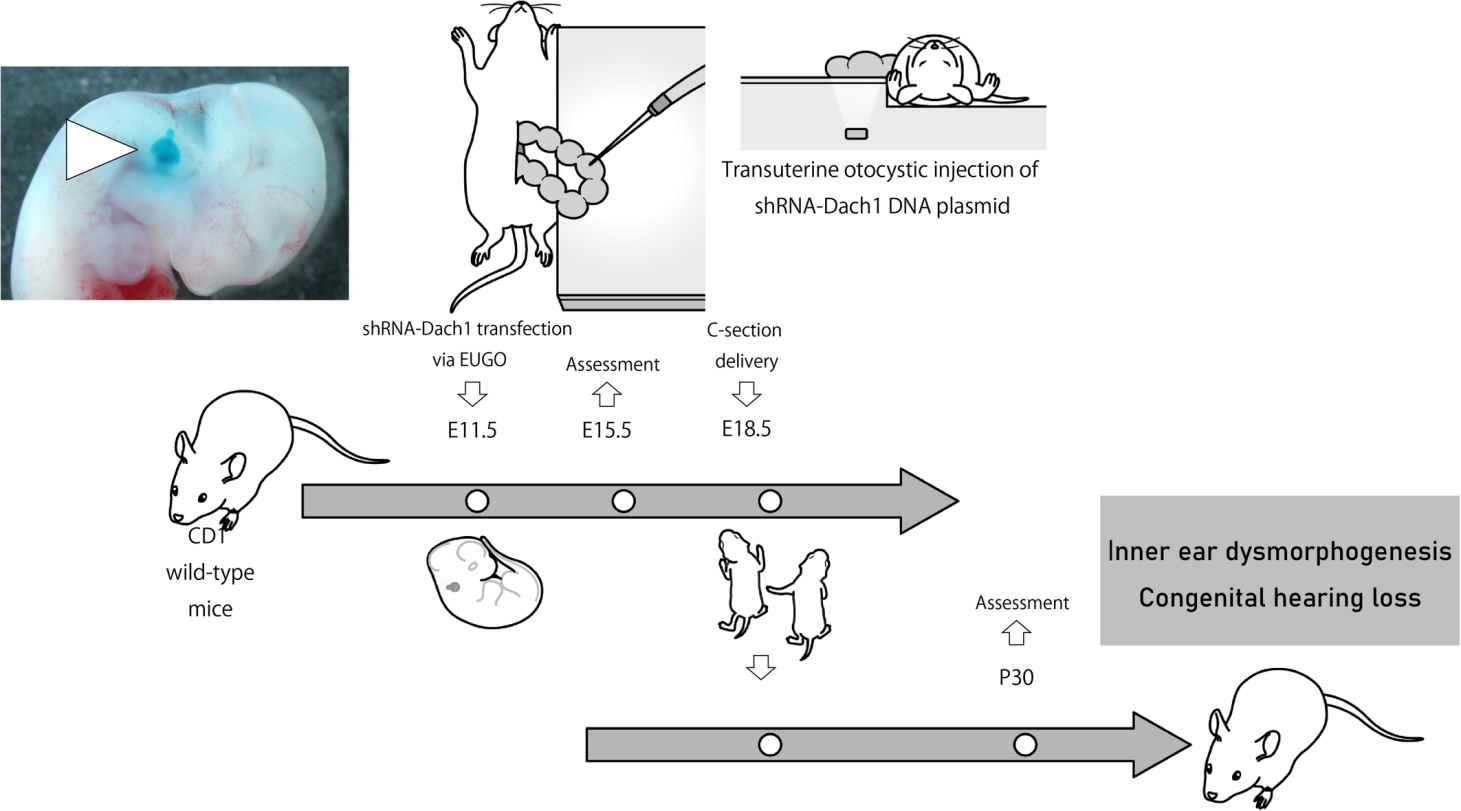
**Novel inner ear specific gene knockdown system.** Electroporation-mediated transuterine gene transfer into otocysts (EUGO) was performed in mice at E11.5. Embryos were delivered via C-section at E18.5, and the pups that underwent gene transfer at E11.5 were passed to surrogate dams.

**What's next for you?**

Now, I am finalizing other developmental research regarding congenital hearing loss and trying to plan for the ENT and researcher life after the post-doctoral position. Preferably, I will pursue a principal investigator position in academia.

## References

[BIO046821C1] Miwa T., Minoda R., Ishikawa Y., Kajii T., Orita Y. and Ohyama T (2019). Role of Dach1 revealed using a novel inner ear-specific Dach1-knockdown mouse model. *Biology Open* 8, bio043612 10.1242/bio.043612 31405829 PMC6737983

[BIO046821C2] Miwa T., Minoda R., Ise M., Yamada T. and Yumoto E. (2013). Mouse otocyst transuterine gene transfer restores hearing in mice with connexin 30 deletion-associated hearing loss. *Mol. Ther.* 21, 1142-1150. 10.1038/mt.2013.62 23587925 PMC3677317

